# How the brain predicts timing: distinct network hubs for predicting and evaluating auditory sensory events

**DOI:** 10.3389/fnins.2026.1739294

**Published:** 2026-04-07

**Authors:** Péter Nagy, Petra Kovács, Ádám Boncz, Orsolya Szalárdy, Robert Baumgartner, Karolina Ignatiadis, István Winkler, Brigitta Tóth

**Affiliations:** 1 Institute of Cognitive Neuroscience and Psychology, HUN-REN Research Centre for Natural Sciences, Budapest, Hungary; 2 Department of Artificial Intelligence and Systems Engineering, Faculty of Electrical Engineering and Informatics, Budapest University of Technology and Economics, Budapest, Hungary; 3 Department of Cognitive Science, Faculty of Natural Sciences, Budapest University of Technology and Economics, Budapest, Hungary; 4 Acoustics Research Institute, Austrian Academy of Sciences, Vienna, Austria; 5 Institute of Behavioral Sciences, Faculty of Medicine, Semmelweis University, Budapest, Hungary

**Keywords:** auditory perception, brain network hubs, EEG, functional connectivity, Normalized Directed Transfer Entropy (NDTE), predictive processing, sensory evaluation, temporal prediction

## Abstract

**Introduction:**

Temporal prediction enhances perceptual processing by aligning neural excitability with expected sensory events. While local oscillatory mechanisms are known to support timing, less is understood about how large-scale functional brain networks dynamically coordinate predictive processes. In particular, it remains unclear how functional connectivity (FC)–the integration of information into network hubs–differs during expectation formation (prediction) versus post-target outcome evaluation, and how this varies across levels of predictability.

**Methods:**

To investigate this, we recorded electroencephalographic data (EEG) while participants performed a cued auditory target-detection task with varying temporal predictability (80% and 50%). FC was analyzed using a data-driven approach based on Normalized Directed Transfer Entropy (NDTE) applied to EEG difference waveforms between high- and low-predictability conditions, separately for the post-cue and post-target periods to distinguish prediction and evaluation phases.

**Results:**

Behaviorally, higher temporal predictability facilitated faster reaction times. Event-related potential (ERP) results revealed that implicit temporal predictability primarily modulated later evaluative processes (P3b, frontal negativity), rather than early sensory components, consistent with context updating under uncertainty. FC analyses revealed that the fronto-temporo-parietal network engaged in the prediction phase evolves into a more focal auditory–frontal circuit during the evaluation of the prediction outcomes.

**Discussion:**

Our findings highlight that temporal prediction and evaluation are supported by the dynamic interactions among multiple large-scale networks rather than by any single region or pathway, supporting both frontal-dominant and distributed integration models of predictive processing.

## 1 Introduction

To navigate the world efficiently, the brain must not only recognize what is happening, but also anticipate when it will happen. Temporal prediction–the ability to forecast the timing of sensory events–enables the nervous system to align moments of heightened neural excitability with expected stimuli, optimizing perception and behavior (Jones, 2018; Large and Jones, 1999; Nobre and van Ede, 2018). When the timing of stimuli is predictable, behavioral responses improve (e.g., faster reaction times, increased accuracy; Toosi et al., 2017), suggesting that predictive timing modulates neural dynamics to prioritize the relevant sensory input. Although local cortical oscillations in delta/theta bands support the processes of temporal prediction (Schroeder and Lakatos, 2009; Arnal and Giraud, 2012; Benedetto et al., 2021), less is known about how timing signals are communicated within large-scale brain networks. The present study addresses this gap by investigating how large-scale networks of cortical hubs dynamically coordinate during different phases of temporal prediction.

A growing body of research suggests that the mechanisms underlying temporal prediction involve both local oscillatory dynamics and interactions across large-scale brain networks. Cortical oscillations–particularly in the delta (0.5–4 Hz) and theta (4–8 Hz) bands–regulate temporal expectations by modulating excitability (Schroeder and Lakatos, 2009; Arnal and Giraud, 2012; Benedetto et al., 2021). For instance, the phase of low-frequency oscillations resets before an anticipated stimulus, and stronger phase alignment has been observed for temporally predictable events (Lakatos et al., 2008; Stefanics et al., 2010). These local entrainment processes are foundational for timing, but they do not fully explain how timing-related information propagates and is integrated across the brain.

Temporal prediction also engages large-scale functional connectivity (FC) networks that coordinate sensory and higher-order cortical regions (Arnal et al., 2015; Darriba and Waszak, 2018). Defined as the synchronization or statistical dependence of activity between distant brain regions, FC supports the flow of predictive information, possibly enabling top-down modulation from higher-order to sensory areas (Sauseng and Klimesch, 2008). While previous research has implicated both frontal and sensory cortices in temporal prediction (Coull et al., 2016; Meirhaeghe et al., 2021; de Lange et al., 2018), the specific architecture and directional roles of network hubs–especially their dynamic involvement during different temporal prediction phases–remain unresolved. Whether predictive timing signals originate predominantly in frontal hubs or emerge within sensory regions (Rao and Ballard, 1999; Kok et al., 2012; Alamia and VanRullen, 2019), or whether they are distributed across both (Hohwy, 2013; van Moorselaar and Slagter, 2019) is unclear.

A parallel line of research, reviewed by Korka et al. (2022), demonstrates that intention-based prediction has a strong influence on auditory processing–even in passive situations and for stimulus-omission paradigms–supporting the notion of the Auditory Event Representation System (AERS; Winkler and Schröger, 2015). Korka et al.’s (2022) extended AERS model illustrates how action intentions elicit predictive signals transmitted from the frontal to the auditory cortices, operating from pre-attentive (e.g., MMN) to later evaluative stages. Critically, intention-generated predictions can override sensory regularities, emphasizing top-down influences on hierarchical processing. These findings raise the question: do intention- and stimulus-based predictions rely on a single common or multiple distinct network hubs, and are they active before or after target onset?

There is moreover an ongoing theoretical debate regarding whether temporal predictions originate in higher-order frontal regions and are relayed to sensory cortices (e.g., predictive coding theory; Friston, 2005; Clark, 2013), or whether sensory areas can independently encode and utilize temporal regularities (Rao and Ballard, 1999; Kok et al., 2012; Alamia and VanRullen, 2019). Hybrid models propose a division of labor, with frontal areas providing top-down predictions and sensory cortices generating prediction errors or updating local representations (Hohwy, 2013; Nobre and van Ede, 2018; van Moorselaar and Slagter, 2019).

It is still largely unresolved how predictive and evaluative processes are dynamically organized within large-scale brain networks. Most electrophysiological studies focus either on local oscillatory mechanisms or on averaged connectivity patterns, without explicitly separating the formation of temporal expectations from the subsequent evaluation of sensory outcomes. As a result, prediction-related and outcome-related processes are often conflated, making it difficult to determine whether the same cortical hubs support both phases or whether network architecture reorganizes once sensory evidence becomes available. Moreover, few studies address the directionality of information flow across these phases, which is critical for distinguishing top-down predictive signaling from bottom-up evaluative updating.

Therefore, in the current study, we aim to test whether temporal prediction and sensory evaluation rely on distinct or overlapping large-scale network hubs, and whether the direction of information flow changes across these phases. We distinguish the prediction phase, during which temporal expectations are formed and maintained following a cue from the evaluation phase, during which incoming sensory evidence confirms or violates those expectations. This distinction allows us to investigate how predictive timing and feedback mechanisms evolve over time and whether the same or different network hubs are engaged at each stage.

To this end, we used a cued auditory detection task with manipulated temporal predictability. Participants heard a cue that probabilistically predicted the timing (early or late) of an upcoming target sound. Based on this design, we defined two conditions corresponding to the predictive value of cues: in the 80% predictable condition, early targets followed the cue with high probability (80%), whereas in the 50% predictable condition, the cue provided no reliable timing information (early targets followed the cue with 50% probability). We hypothesized that early targets preceded by predictive cues (predicting the target with 80% probability) would elicit faster reaction times (RTs) than early targets preceded by non-predictive cues (predicting the target with 50% probability). By applying a directed, hub-based functional connectivity framework to post-cue and post-target intervals, we aimed to reveal how predictive brain networks dynamically reorganize across time with two competing hypotheses in mind: (1) a frontal-dominant account, in which predictive signals converge on frontal hubs during expectation formation and propagate toward sensory regions during evaluation (consistent with hierarchical predictive coding see Friston, 2005; Alamia and VanRullen, 2019), and (2) a distributed integration account, in which predictive and evaluative information is more symmetrically integrated across frontal and sensory cortices (Kok et al., 2012; Benedetto et al., 2021).

## 2 Materials and methods

### 2.1 Participants

Twenty healthy young adults participated in the study (10 female; mean age: 22.4 ± 2.35 years, all right-handed). Participants were financially compensated for their participation (approx. 4 euros per hour). None of the participants reported any neurological diseases or hearing problems. Participants signed written informed consent forms after the aims and procedures of the experiment were explained. The study adhered to the Declaration of Helsinki guidelines and was approved by the local ethics committee of the Institute of Cognitive Neuroscience and Psychology at the HUN-REN Research Center for Natural Science (United Ethical Review Committee for Research in Psychology; EPKEB). The EEG data of 3 participants were excluded from the analysis due to technical issues (missing blocks) during data acquisition. The final group for which EEG analysis was conducted consisted of 17 listeners (10 female and 7 male), with a mean age of 22.6 ± 2.45 years.

### 2.2 Stimuli and procedure

**FIGURE 1:**
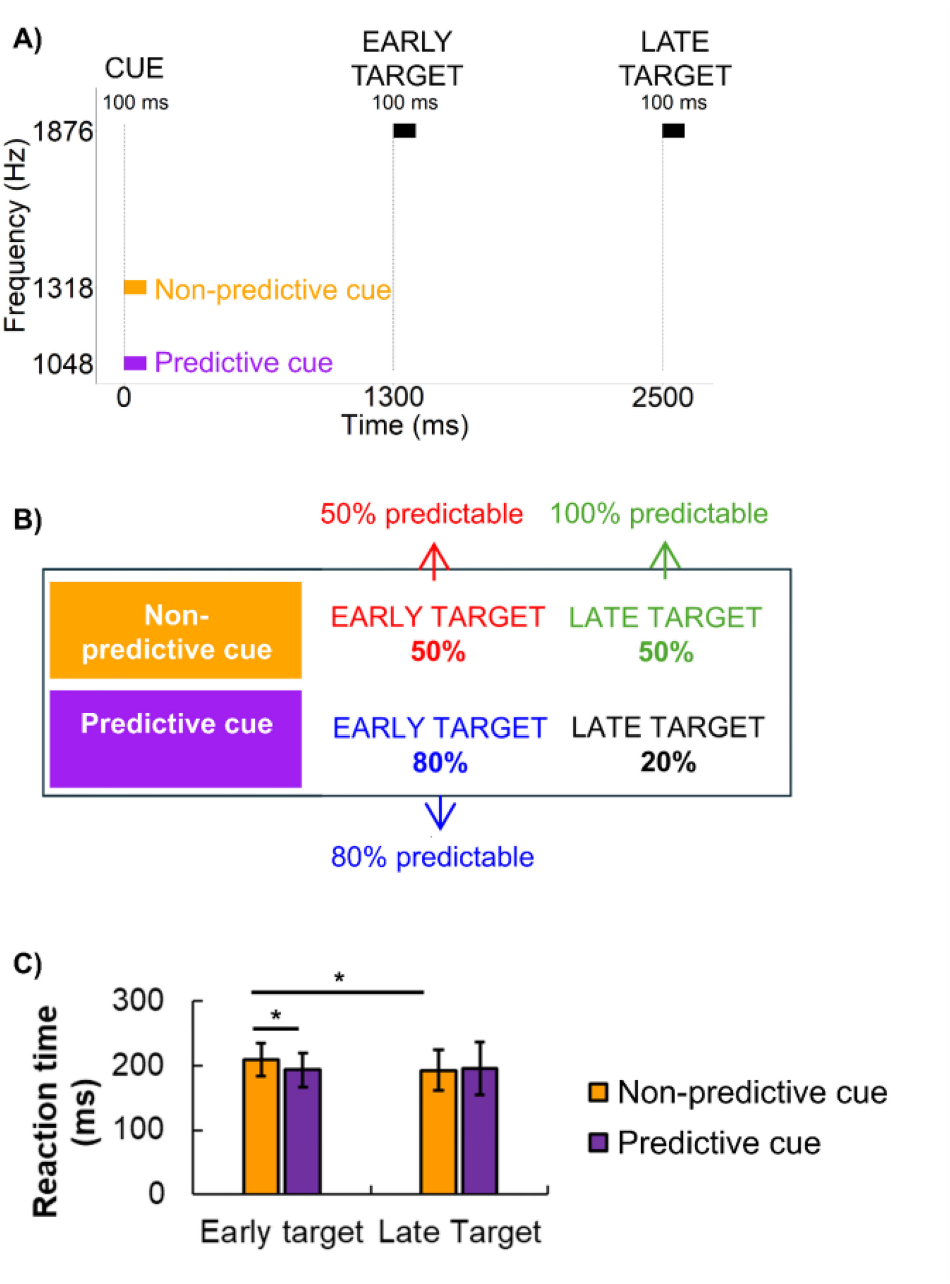
Stimuli, conditions, and reaction time results. **(A)** Timeline of the stimulus presentation. On each trial, one cue and one target were presented, each lasting 100 ms. The target followed the cue after either a short or a long cue-target-interval (early vs. late target). Two types of cues were used: non-predictive and predictive cues. The cues differed in their pitch. **(B)** After a predictive cue, an early target followed with 80% certainty, and after a non-predictive cue, early and late targets followed with equal likelihood. We refer to the 50%, 80%, and 100% within-trial predictability, based on their within-trial probability, as shown in the figure. **(C)** Mean RTs to the target events and the results of the comparisons between them (horizontal lines with an asterisk above them denoting significant differences; error bars denote standard deviation).

Participants performed an auditory target detection task while EEG was recorded. They were instructed to detect target tones as quickly as possible by pressing a key on a computer keyboard with the index finger of their dominant hand (all participants were right-handed). Each target was preceded by an auditory cue (Figure 1A).

The interval between the cue and the target was either 1200 or 2400 ms, corresponding to early and late targets, respectively. The inter-trial interval (ITI) varied randomly between 200 and 1000 ms to minimize temporal predictability of the onset of the next trial. All stimuli were presented at a uniform sound pressure level of approximately 70 dB (SPL).

Two distinct cue tones were used to manipulate the predictability of the target’s timing. These tones had frequencies of 1048 and 1318 Hz, each presented for 100 ms with 10 ms linear rise and fall times. The target tone was always 1876 Hz, with a duration of 100 ms, including 10 ms rise and fall times. The cue frequencies were separated by one semitone, while the target tone was two semitones above the higher cue, ensuring clear perceptual distinction.

One cue served as a predictive cue, while the other served as a non-predictive cue, with the assignment counterbalanced across participants (Figure 1B). After the predictive cue, the target was followed with a 1200 ms long cue-target-interval on 80% of the trials, making early targets more likely than late ones. In contrast, the non-predictive cue was followed by an early or a late target with equal probability (50%). Participants were not informed about the predictive values of the cues. Based on this design, we identified three levels of temporal predictability for target events: (1) 50% predictable: early targets following a non-predictive cue. (2) 80% predictable: early targets following a predictive cue. (3) 100% predictable: late targets, which occurred with certainty (100%) if an early target did not appear after the cue. Each participant completed three blocks of 50 trials per condition (predictive cue or non-predictive cue), resulting in a total of 300 target trials (150 per condition). The order of the four types of trials (80% predictive cue followed by an early target; 80% predictive cue followed by a late target; 50% predictive cue followed by an early target; 50% predictive cue followed by a late target) was randomized across participants. All 4 types of trials appeared within the same block of trials. Before the main experiment, participants completed a brief familiarization session with the stimuli and task.

We examined how predictability modulates neural and behavioral responses. However, since the targets with 100% predictability were presented after a longer delay, they were not directly comparable to the early targets, due to differences in response selection, preparatory processes, and movement execution. Thus, the 100% predictable targets were considered only in the behavioral analysis, whereas neural analyses focused solely on the 50% and 80% predictable early targets. This ensured conceptual alignment between behavioral and neural components of the study while preserving continuity with the foundational behavioral design.

### 2.3 EEG recording and preprocessing

For EEG recording, a BrainAmp DC 64-channel EEG system with actiCAP active electrodes was used. The sampling rate was 1 kHz, with a 100 Hz online low-pass filter applied. Electrodes were placed according to the international 10/20 system. The FCz channel served as the reference electrode. Two electrodes placed laterally to the eyes’ outer canthi monitored eye movements. During the recording, impedances were kept below 15 kΩ.

Electroencephalographic data preprocessing was conducted with the EEGLAB 21.1 toolbox (Delorme and Makeig, 2004) implemented in MATLAB (2021b, Mathworks, Natick, Massachusetts, USA). Offline band-pass filtering was applied between 0.05 and 80 Hz using a low-pass/high-pass filter cascade of Hamming-windowed sinc filters (EEGLAB pop_eegfiltnew, zero-phase non-causal filter, high-pass filter order = 66000, low-pass filter order = 166). The data were then re-referenced to the average of all EEG channels. The Infomax algorithm for independent component analysis (ICA) with principal component analysis (PCA)-based dimensionality reduction (PCA dimension = 32) was employed for artifact removal (Delorme et al., 2007). ICA components constituting blink artifacts were removed after a visual inspection of their topographical distribution and the frequency contents of the components. A maximum of 3 components per participant was removed in this way (<10%). Malfunctioning channels were identified based on channel spectral characteristics and ICA components. A maximum of 6 malfunctioning EEG channels per participant was allowed without exclusion from the study. These EEG channels were interpolated using the default spline interpolation algorithm implemented in EEGLAB. Then, the data were low-pass filtered (using a Hamming-windowed sinc filter with a 45 Hz cutoff frequency, a zero-phase, non-causal filter, and a filter order of 294) to attenuate high-amplitude transient noise peaks, and epochs between −500 and +4500 ms were extracted relative to the onset of cue events. Epochs including an amplitude change of 100 μV or greater were rejected from further analysis (ratio of rejected epochs: *M* = 9.7%, SD = 10.1%). Because the number of epochs for early targets in the 80% predictable condition was higher than that for early targets in the 50% predictable condition, the number of epochs was equalized between these trial types in a within-participant manner. To this end, early-target epochs were randomly removed (Matlab *randperm* function) from the 80% predictable condition, ensuring the number of remaining epochs was equal to that in the 50% predictable condition for the same participant. Thus, the number of epochs varied across participants but was consistent across the two critical trial types (*M* = 69.12; SD = 5.06).

### 2.4 Data analysis

#### 2.4.1 Behavioral data

Statistical analyses were performed in STATISTICA (version 13.1, TIBCO Software Inc., Palo Alto, California, USA). A 2 × 2 repeated-measures ANOVA was used to compare the effects of condition (80% predictable vs. 50% predictable) and target type (early vs. late) on reaction time (RT) measured from target onset. Response times longer than 1000 ms from target onset were excluded from the analysis. The alpha level was set at 0.05. Partial eta squared (${\text{η}}_{\text{p}}^{2}$) is reported as the measure of effect size. *Post-hoc* tests were computed by Tukey’s “Honest Significant Difference” (HSD) pairwise comparisons (Tukey, 1949). This approach replicates the analysis used in Stefanics et al. (2010), whose paradigm we closely followed in terms of stimulation and temporal structure. Including both early and late targets in the behavioral analysis allowed us to confirm the presence of cueing effects across the full range of target probabilities, providing a behavioral benchmark for comparability with prior work.

#### 2.4.2 ERP cluster analysis

After preprocessing, ERP cluster-based permutation analysis was applied to compare cue- and target-evoked potentials across the two conditions (50% and 80% predictable). The cluster-based analysis was performed using the Brainstorm MATLAB toolbox (Tadel et al., 2011; version 11 July 2024). For each comparison, a permutation test was applied to the epochs. In the post-cue period, epochs for the ERP permutation test were defined from 0 to 1300 ms relative to cue onset (i.e., the entire period between cue onset and the subsequent target onset). In the post-target period, epochs for the ERP permutation test were defined between 0 and 1300 ms relative to the onset of early target events to maintain comparability with the post-cue period. The conditions were compared with two-tailed paired *t*-tests using 1000 permutations. The minimum number of neighboring channels was set to 3, and the alpha level was set to 0.05.

#### 2.4.3 EEG source localization and functional connectivity analysis

The Brainstorm toolbox (Tadel et al., 2011; version 11 July 2024) was used to perform EEG source reconstruction, following the protocol of previous studies (Song et al., 2015; Huang et al., 2016; Pizzagalli, 2007; Tóth et al., 2020). The forward boundary element method (BEM) head model was used as provided by the openMEEG algorithm (Fuchs et al., 1998, 2002; Gramfort et al., 2010). Individual structural MRI and electrode positions were available for each participant and used for head modeling to improve EEG source localization accuracy (Ignatiadis et al., 2022). Anatomical magnetic resonance images (MRIs) were segmented via Freesurfer (Fischl, 2012). The recorded activity was mapped onto the cortical surface via dynamic statistical parametric mapping (dSPM, Dale et al., 2000). The noise covariance was calculated from a 200-ms interval preceding the onset of cue events. Dipole orientations were considered as constrained to the surface, and source signals were reconstructed at 15000 vertices describing the pial surface. By averaging dipole strengths across voxels, the mean source waveforms were obtained for 62 cortical areas (regions of interest, ROIs) based on the anatomical segmentation of the Desikan-Killiany-Tourville atlas (DKT atlas, Klein and Tourville, 2012). Mean source waveforms corresponding to the 62 ROIs were used for FC analyses.

We followed a common two-stage EEG analysis strategy, using ERP results to identify relevant time intervals and then analyzing FC. The post-target window (100–500 ms after target onset) was defined based on a significant ERP cluster reflecting evaluative processes (see Section “3 Results”). Because the specified window was 400 ms, the reconstructed ROI activities were high-pass filtered at 2.5 Hz to retain the period of the slowest oscillation allowed by the window length. No significant ERP effects were found in the post-cue period (see Section “3 Results”), consistent with evidence that implicit temporal prediction involves slow, distributed oscillatory activity rather than focal evoked responses. We therefore selected the final 400 ms before target onset as an *a priori* window, motivated by theories of temporal anticipation: During that period, the anticipatory process is assumed to build gradually and continuously (Walter et al., 1964; Kononowicz and Penney, 2016). Based on this notion, the last 400 ms of the post-cue period (between 900 and 1300 ms after cue onset) was used to maintain comparability with the post-target period. Similarly, the same high-pass filtering (2.5 Hz) was applied as in the post-target period. While defined asymmetrically, the windows reflect distinct neural dynamics and adhere to established analytic practice.

Finally, epochs were paired by conditions (one epoch was selected from the 80% predictable condition, the other epoch was selected from the 50% predictable condition). Epochs were paired randomly, the randomization was done separately for each participant (Matlab *randperm* function). For each epoch pair, the waveforms were extracted for each ROI, and the 50% predictable − 80% predictable difference waveform was calculated. These difference waveforms were used for FC analyses.

For the FC analysis, we applied a hub-based connectivity approach. In this approach, brain regions of interest (ROIs) constitute a network: ROIs are the nodes of the network and functional connections between ROIs are the edges of the network. Highly connected ROIs are commonly referred to as hubs. Deco et al. (2021) proposed the concept of a functional rich club (FRIC) as the core set of ROIs, an array of functional hubs that are characterized by a tendency to be more densely functionally connected among themselves than to other ROIs from where they receive information. The FRIC typically receives more information (information “inflow” to the FRIC) from outside ROIs than the amount of information they send/communicate to ROIs outside. FC was analyzed using the Normalized Directed Transfer Entropy (NDTE) framework (Deco et al., 2021; see Ignatiadis et al., 2025, for an application on EEG data). The NDTE framework provides a bidirectional (inflow/outflow) description of the functional information flow. The NDTE flow F_XY_ from time series X to Y (from a source brain area X to another target brain area Y) is defined as the mutual information corresponding to the degree of statistical dependence between the past of X and the future of Y, normalized by the joint mutual information that the past of both signals together, X and Y, have about the future of Y. The normalization enables one to compare and combine the directed mutual information flow across different pairs of brain regions. The mutual information can be calculated from conditional entropies, and the conditional entropies can be estimated from the covariance matrices of X and Y (Brovelli et al., 2015). Following Deco et al. (2021), we estimated the order (or “maximum lag”) of the NDTE model by fitting the autocorrelation function to the first minimum across conditions and participants. The resulting average decay times were 15.09 ± 4.19 ms for the post-cue period and 14.56 ± 2.62 ms for the post-target period. These values determined the integration time window used to estimate directed information flow between regions.

Normalized Directed Transfer Entropy flow values were tested for statistical significance and standardized using circular-shift surrogate data following the method proposed by Deco et al. (2021, 2022). The advantage of time-shifted surrogates is that the properties (e.g., amplitude and autocorrelation spectrum) of each signal are maintained because the signals are the same, but the cross-correlation between the signals is reduced to chance level (Quiroga et al., 2002). Following the method proposed by Deco et al. (2021), 100 independent circular time-shifted surrogate iterations were performed for each considered ROI pair in each trial. Statistical significance of connections between ROIs was calculated using *p*-value aggregation via Stouffer’s method (Stouffer et al., 1949) across trials within a participant, and subsequently across all participants. After aggregating *p*-values across trials and participants, the correction for multiple comparisons was performed using the false discovery rate (FDR) method of Benjamini and Hochberg (1995). The corrected values were then used to select significant connections: one binary significance mask of dimensions ROI × ROI was used for all participants, separately for the post-cue and post-target period. Finally, significant NDTE flow values were standardized by subtracting the mean and dividing by the standard deviation of the corresponding 100 surrogate NDTE flow values. The result is one NDTE matrix of dimensions ROI × ROI (inflow ROIs positioned along the first dimension, outflow ROIs positioned along the second dimension) for each subject in each epoch, both in the post-cue and in the post-target period, containing the standardized NDTE flow values for the ROI pairs that survived the significance test. The NDTE matrix can be considered a network: ROIs are the nodes of the network, standardized NDTE flow values are the directed connections (edges) of the network (an inflow connection represents information incoming to an ROI, an outflow connection represents information outflowing from an ROI).

Standardized NDTE flow values surviving the significance test were used to identify functional hub regions in the brain. Following the concept of the FRIC, we aimed to determine the core set of brain regions that are more functionally densely connected among themselves than to other brain regions. As the first step in the FRIC analysis, standardized NDTE flow values were averaged across trials within a participant, and subsequently across all participants, yielding a single standardized NDTE matrix of dimensions ROI × ROI both in the post-cue and in the post-target interval. For each ROI, the total inflow from all ROIs of the cortical parcellation is defined as the sum of connectivity across all columns of the matrix. The total outflow per ROI is defined as the sum of connectivity across all rows of the matrix.

The major hubs were defined through an iterative process. After sorting the regions by inflow from highest to lowest, the largest subset of ROIs was identified that had a significantly larger FRIC-value than any other set with the same number of regions. The FRIC-value is defined as the sum of connections between all club members, plus the sum of all inflow connections to the club members, minus the sum of all outflow connections of the club members. The significance of the FRIC-value corresponding to a club with k members was assessed via 100000 Monte Carlo simulations for each tested value of k. In each permutation, surrogate clubs with k members were created, each having the same k−1 members and one additional random member. Starting with the ROI with the largest inflow (the sum of all inflow connections of the given ROI), the algorithm continued to consider larger clubs as long as the *p*-value of the comparison between the considered club and the surrogate clubs was less than 0.05.

In summary, this approach complements traditional node-based or symmetric FC methods by providing a directional view of network dynamics (Fiebelkorn and Kastner, 2019; Ignatiadis et al., 2025). The NDTE framework considers the interconnectivity of all defined brain regions and includes a suitable normalization using time-shifted surrogates and *p*-value aggregation to eliminate spurious inferences. This framework provides a robust, data-driven approach for investigating functional hubs of brain networks without the need for a-priori defining brain regions or constraining the structure of the network. The mathematical formulations of the NDTE framework and the FRIC method, as proposed by Deco et al. (2021), are presented in the Appendix.

In our study, the NDTE framework was applied to the 50% and 80% predictable conditions separately, as well as to single-trial difference waveforms (50% predictable − 80% predictable difference waveforms). FRIC hubs corresponding to the 50% and 80% predictable conditions represent the absolute neural information transfer in the baseline system whereas the results obtained for difference waveforms quantify the temporal structure of condition differences that were the focus of our investigation. Ignatiadis et al. (2025) introduced this analysis approach based on difference waveforms to make best use of the surrogate-based inference testing described above and circumvent difficulties of quantitative comparisons between separate results of the selective analysis pipeline. Nevertheless, qualitative comparisons to the individual-condition results were performed to judge the validity of this approach. Functional connections estimated based on single-trial difference waveforms will be referred to as connections of the directed temporal structure of the condition effect.

The brain networks were visualized with the BrainNet Viewer (Xia et al., 2013).^1^

#### 2.4.4 Correlating reaction time differences with functional connectivity

Reaction time differences between the 50% predictable and 80% predictable conditions were correlated with each of the strongest 20 functional inflow connections of the directed temporal structure of the condition effect (∼0.5% of all connections) using Pearson’s correlation coefficient. The correlation was calculated for functional inflow connections in both the post-cue and post-target periods.

#### 2.4.5 Cue-related modulation of neural entrainment

The effect of cue-related modulation on neural entrainment was investigated in *post hoc* analyses. In these analyses, the average bandpower, intertrial phase clustering (ITPC), and phase-based connectivity [phase locking value (PLV), Mormann et al., 2000] of delta-band and broad-band EEG signals in the post-cue period were compared between the 80% and 50% predictable conditions. The time period of cue presentation was excluded from the analysis (the time period between 100 and 1300 ms after cue onset was included). These additional analyses were motivated by prior work suggesting that low-frequency entrainment may still be functionally relevant even when subthreshold in scalp averages (Lakatos et al., 2008; Stefanics et al., 2010).

Average bandpower was estimated for each EEG channel and for the average of all EEG channels by Power Spectral Density (PSD) using Welch’s method (segments with 50% overlap, frequency resolution of 0.12 Hz) averaged in the delta band (0.5–4 Hz) and broad band (0.5–80 Hz). Average bandpower values estimated in the post-cue period were normalized by average bandpower values estimated during the baseline period. Normalized average bandpower values were compared between the 50% and 80% predictable conditions using non-parametric permutation tests (1000 iterations, Maris and Oostenveld, 2007). An alpha level of 0.05 was used. Significance values were corrected against multiple comparisons using the Benjamini-Hochberg method (Benjamini and Hochberg, 1995).

To estimate ITPC, time-frequency decomposition of EEG data was performed by convolving complex Morlet wavelets with EEG data. Center frequencies of the wavelets were logarithmically spaced between 0.5 and 80 Hz in 20 steps. ITPC values were averaged across frequency within the frequency bands of interest (0.5–4 Hz and 0.5–80 Hz). Finally, cluster-based analysis was performed on ITCP values using the Brainstorm MATLAB toolbox. The conditions were compared with two-tailed paired *t*-tests using 1000 permutations. The minimum number of neighboring channels was set to 3, and the alpha level was set to 0.05.

Phase-based connectivity between EEG channels was estimated using the PLV measure (Mormann et al., 2000). PLV measures the magnitude of the mean phase difference between the two signals, with phase differences expressed as complex unit-length vectors. The discrete-time PLV is defined as follows:


$$P\,L\,V=\left|\frac{1}{T}\,\overset{T}{\underset{t=1}{\sum }}e^{j\,\left(\phi_{a}\,\left(t\right)-\phi_{b}\,\left(t\right)\right)}\right|$$

where *T* is the data length, Φ_*a*_ and Φ_*b*_ are the instantaneous phases of the signals *s*_*a*_ and *s*_*b*_. The instantaneous phase of an arbitrary signal *s(t)* can be expressed as


ϕ⁢(t)=arctan⁢s(t)∧s⁢(t)

where s(t)∧ is the Hilbert transform of the signal *s(t)*.

Phase locking values of all possible channel pairings were averaged resulting in one whole-head average PLV value for each epoch and participant, then these values were averaged across epochs. For PLV estimation, EEG was high-pass filtered at 0.85 Hz considering the 1200 ms epoch length (the frequency bands of interest were 0.85–4 Hz and 0.85–80 Hz in this analysis). Subject-level average PLV values were compared between the 50% and 80% predictable conditions using non-parametric permutation tests (1000 iterations). An alpha level of 0.05 was used.

## 3 Results

### 3.1 Behavioral results

The task design included three target types based on their within-trial predictability: (1) 50% predictable targets: early targets following an non-predictive cue, (2) 80% predictable targets: early targets following a predictive cue, and (3) 100% predictable targets: late targets, which occurred if an early target was absent (Figures 1A, B).

A repeated-measures ANOVA was conducted on RTs with two within-participant factors: CONDITION (80% predictable vs. 50% predictable) and TARGET TYPE (early vs. late). The analysis revealed a significant interaction between CONDITION and TARGET TYPE, *F*(1,19) = 11.20, *p* < 0.01, ${\text{η}}_{\text{p}}^{2}$ = 0.37, as well as a significant main effect of CONDITION, *F*(1,19) = 5.92, *p* < 0.05, ${\text{η}}_{\text{p}}^{2}$ = 0.24. *Post hoc* comparisons confirmed that RTs were significantly slower in the 50% predictable condition compared to the 80% predictable condition, *t*(19) = 4.97, *p* < 0.001, Cohen’s *d* = 0.70 (Figure 1C).

As predicted, RTs were significantly faster for 100% predictable targets (late targets) than for 50% predictable targets, *t*(19) = 5.05, *p* < 0.001, Cohen’s *d* = 0.58. In contrast, no significant difference was found between 80% and 100% predictable targets, *p* > 0.05.

### 3.2 ERP results

**FIGURE 2:**
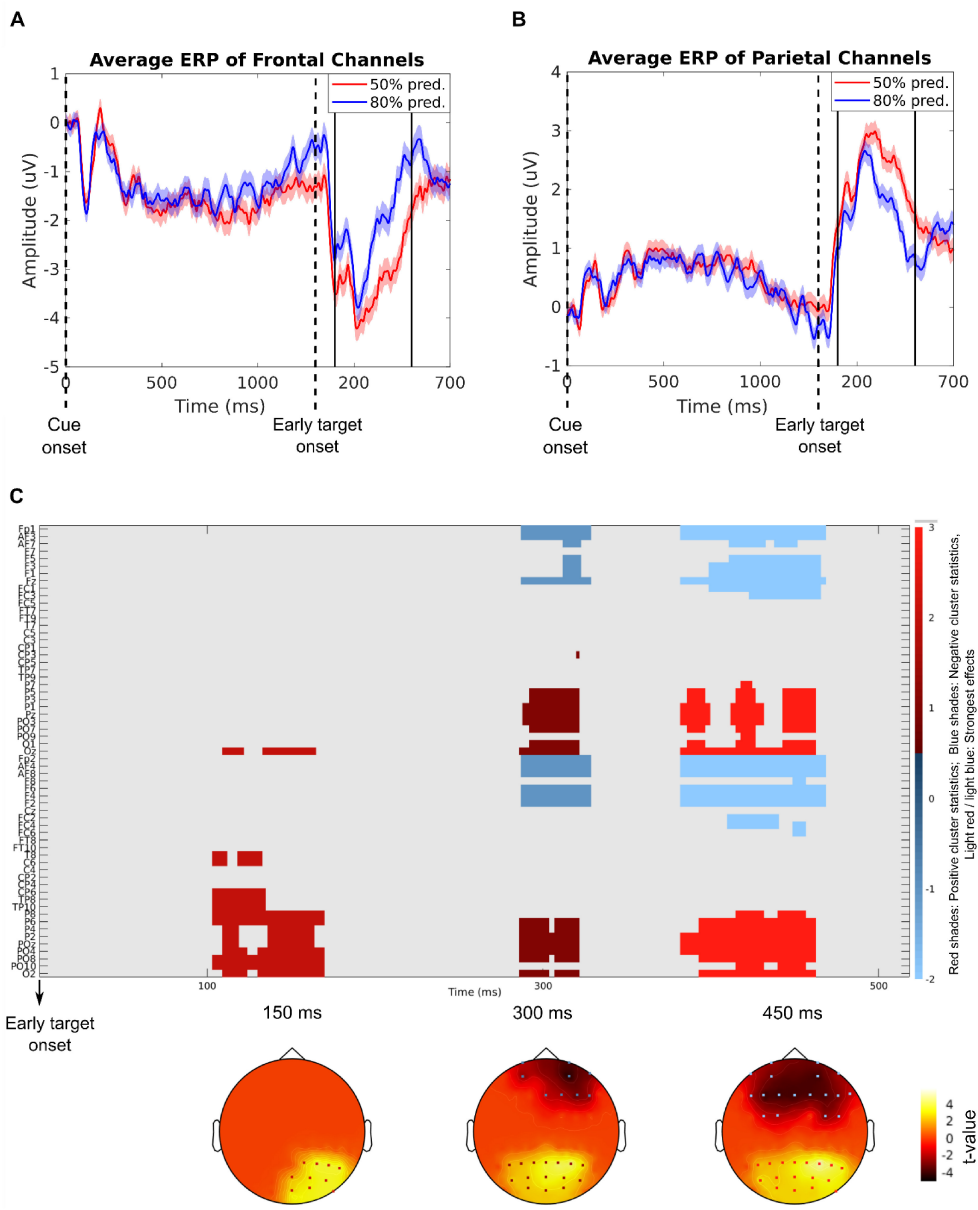
Event-related potentials (ERPs) evoked by the cue and the target stimulus of the trials at the average of the frontal electrodes [Fp1, Fp2, AF3, AF4, AF7, AF8, Fz, F1, F2, F3, F4, F5, F6; **(A)**] and the average of the parietal electrodes [Pz, P1, P2, P3, P4, P5, P6, POz, PO3, PO4, PO7, PO9; **(B)**] showing the most pronounced difference between the 50% predictable (red) and 80% predictable (blue) conditions. Shaded areas denote the standard error. The time range showing the most pronounced difference in the cluster-based permutation test (1400–1800 ms after cue onset) is indicated by black vertical lines. Panel **(C)** shows the results of the cluster-based permutation test on ERPs elicited by 50% predictable vs. 80% predictable conditions (color scale in arbitrary units). The *x* direction represents time in ms (from 1.3 to 1.8 s after cue onset, where 1.3 corresponds to the target onset). The detected clusters span from ∼1.4 s to 1.8 s–ordinate: EEG electrode names. The color scale indicates the direction and strength of the cluster statistics. Red shades: Positive cluster statistics (i.e., 50% predictable > 80% predictable); Blue shades: Negative cluster statistics (i.e., 50% predictable < 80% predictable). The topographical plots show the distribution of *t*-values in the significant clusters at 150, 300, and 450 ms.

Figures 2A, B illustrate the averaged ERPs elicited by the stimuli in the trial from the electrode locations in the frontal and parietal regions (frontal: Fp1, Fp2, AF3, AF4, AF7, AF8, Fz, F1, F2, F3, F4, F5, F6; parietal: Pz, P1, P2, P3, P4, P5, P6, POz, PO3, PO4, PO7, PO9), which contributed most prominently to the significant spatiotemporal clusters shown in Figure 2C.

The cluster-based permutation test comparing the 50% predictable and 80% predictable conditions revealed significant differences in the post-target period. In contrast, no significant clusters were observed in the post-cue interval (from cue to target). A prominent post-target cluster was observed between approximately 100 and 500 ms after target onset (i.e., 1400–1800 ms after cue onset), with distinct topographical patterns. Frontal electrodes (e.g., Fp1, Fz, AF3, F1) showed more negative potentials in the 50% predictable condition, while parietal electrodes (e.g., Pz, PO3, PO7, P4) showed more positive potentials in the same condition.

### 3.3 Functional connectivity results

The NDTE framework was first applied to the 50% and 80% predictable conditions separately, and then to single-trial difference waveforms quantifying the structure of connections between source-localized cortical regions for the EEG difference between early targets following 80% (predictive) and 50% (non-predictive) cues, i.e., the condition effect. Two key time windows were tested: the post-cue period (900–1300 ms after cue onset) and the post-target period (100–500 ms after target onset; 1400–1800 ms after cue onset).

**FIGURE 3:**
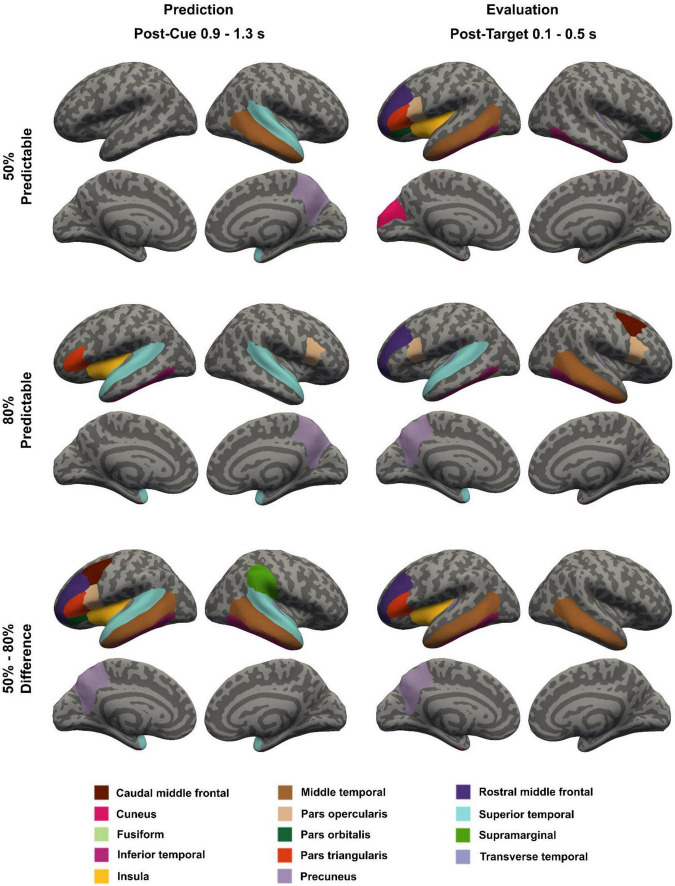
Topography of the cue-predictability related FRIC hubs in the post-cue (0.9–1.3 s from cue onset; left) and post-target intervals (0.1–0.5 s from target onset; right), based on standardized NDTE values averaged over all epochs and participants, in the 50% predictable condition (top), in the 80% predictable condition (middle), and for the 50% predictable – 80% predictable difference waveforms (bottom).

Identified FRIC hubs corresponding to the 50% and 80% predictable conditions and single-trial difference waveforms are shown in Figure 3.

During the target prediction window (900–1300 ms after cue onset), the FRIC analysis of single-trial difference waveforms identified a set of predominantly frontal and temporal regions as hubs (Figure 3, left panel). These included: Frontal - Left rostral middle frontal (rMFG), left caudal middle frontal (cMFG), left pars triangularis, left pars opercularis, left pars orbitalis; Temporal - Bilateral superior, middle, and inferior temporal cortices, right transverse temporal cortex; Other - Left insula, right supramarginal gyrus, and left precuneus. A subset of these hubs were also identified in the 50% and 80% predictable conditions. The right superior temporal cortex was identified in both conditions; the right middle temporal cortex was identified in the 50% predictable condition; the left pars triangularis, left superior and inferior temporal cortices, and left insula were identified in the 80% predictable condition. Other hubs not identified from single-trial difference waveforms but found in the individual conditions included the right pars opercularis and the right precuneus.

In the target evaluation window (100–500 ms after target onset), the FRIC network corresponding to single-trial difference waveforms emerged as a set of predominantly temporal and left frontal regions, which served as hubs (Figure 3, right panel). The hubs included: Frontal - Left rostral middle frontal, left pars triangularis; Temporal - Bilateral middle, and left inferior temporal cortices, left transverse temporal cortex; Other - Left insula, left precuneus. These hubs were also identified in the 50% and 80% predictable conditions. The left rostral middle frontal and left inferior temporal cortex were identified in both conditions; the left pars triangularis, left middle temporal cortex, and the left insula were identified in the 50% predictable condition; the left transverse temporal and right middle temporal cortices, and the left precuneus were identified in the 80% predictable condition. Other hubs not identified from single-trial difference waveforms but found in the individual conditions included the bilateral pars opercularis, left pars orbitalis, right caudal middle frontal cortex, left superior temporal, right inferior and transverse temporal cortices, bilateral fusiform gyri, and the left cuneus.

**FIGURE 4:**
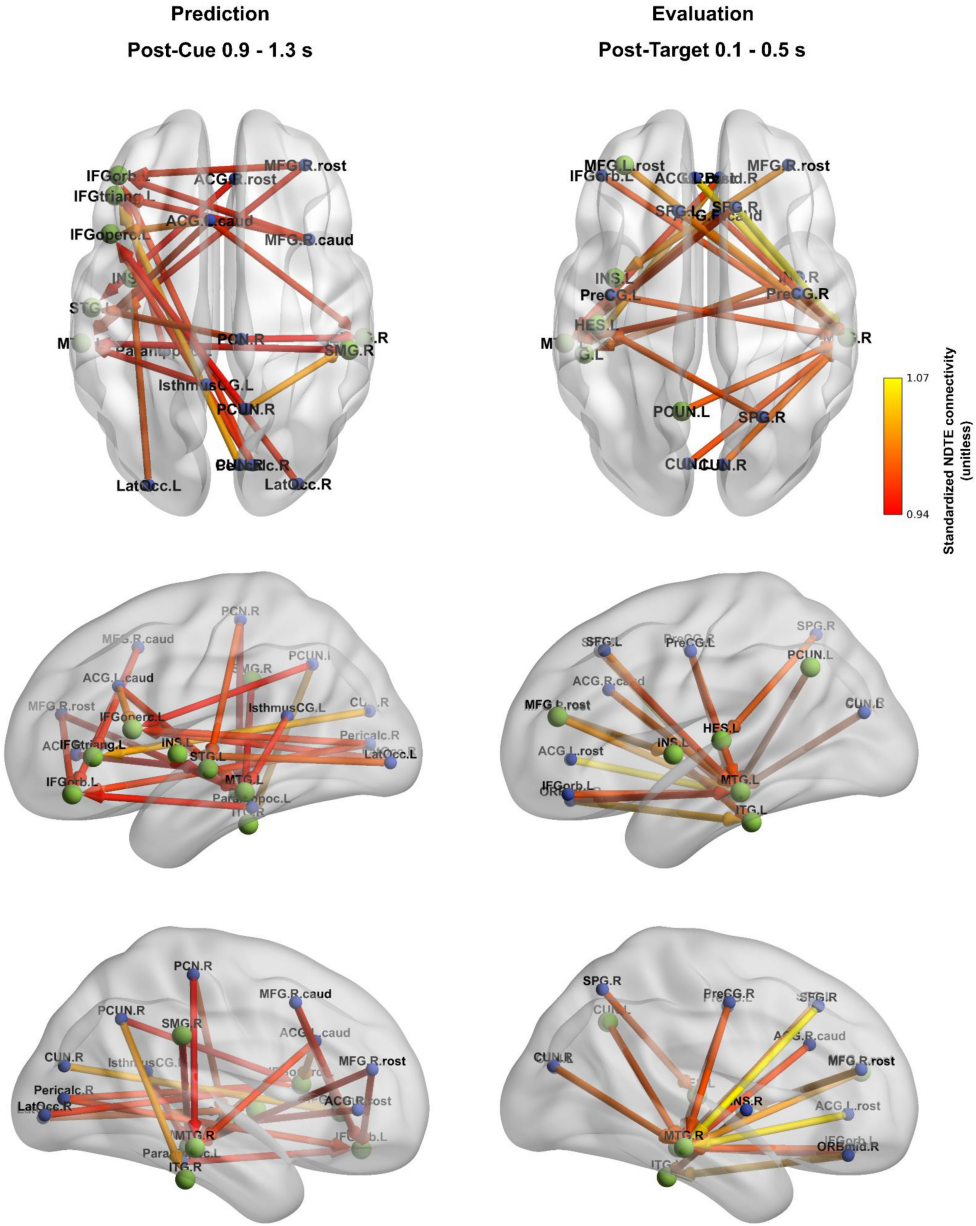
Cue-predictability sensitive functional inflow connections of different brain regions (group-level average). FRIC hubs are colored green and shown with larger node size than ROIs outside the FRIC, which are colored blue. The strongest 20 connections [∼0.5% of all connections] are shown. The color of connections denote standardized NDTE connectivity strength (unitless).

Group-level average inflow connections of the directed temporal structure of the condition effect showing the highest inflow (the strongest 20 connections [∼0.5% of all connections]) are illustrated in Figure 4. These connections are predominantly long-range fronto-temporal, parieto-frontal, and parieto-temporal connections toward FRIC hubs.

The strongest 20 inflow connections during the prediction window (Figure 4, left panel) revealed a predominantly left-lateralized network, with dense inputs to frontal and temporal hub regions. These connections are predominantly interhemispheric. Directed connections prominently connected the middle and superior temporal, as well as the left inferior frontal hubs, to parietal, occipital, and middle frontal areas, consistent with anticipatory processing within the dorsal auditory stream under increased temporal predictability.

During the post-target window (Figure 4, right panel), most inflow paths were directed to the left and right middle temporal as well as left inferior and transverse temporal hubs, receiving information from frontal and parietal regions.

### 3.4 Correlation between Reaction Time Difference and Functional Connectivity

**FIGURE 5:**
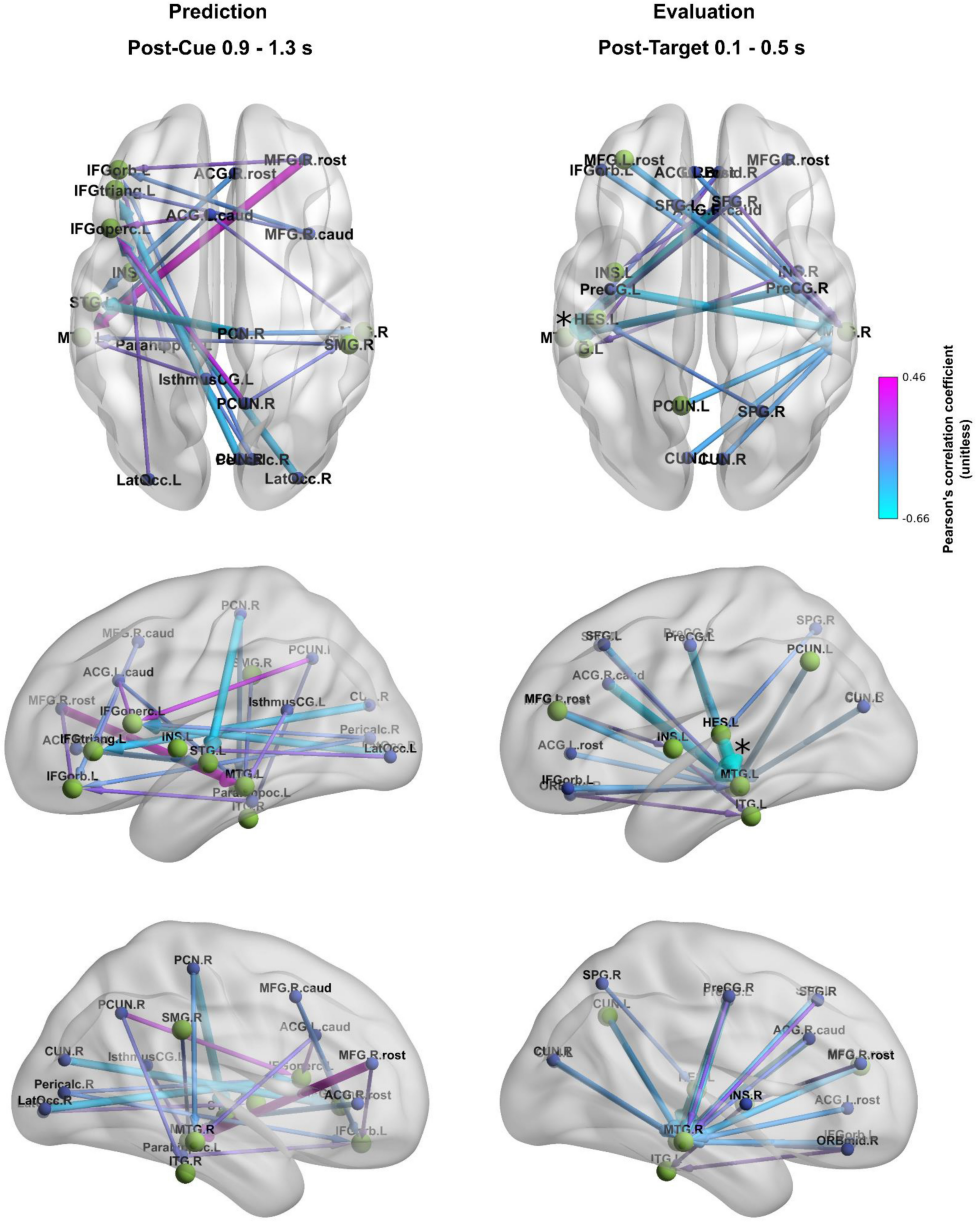
Correlation between the 50% predictable – 80% predictable target reaction time difference and the strongest 20 functional inflow connections (∼0.5% of all connections) in the post-cue (left) and post-target (right) period. FRIC hubs are colored green and shown with larger node size than ROIs outside the FRIC, which are colored blue. The color of connections denotes Pearson’s correlation coefficient (unitless), the width of connections denotes the absolute value of the correlation. The marginally significant correlation is denoted by an asterisk.

The correlation between the 50%-minus-80% predictability condition difference in reaction time and the strongest 20 functional inflow connections of the directed temporal structure of the condition effect was calculated separately for the post-cue and post-target periods (Figure 5). These functional connections differentially correlate with the observed predictability-related reaction time enhancement. A strong relationship between connectivity and behavioral outcome was observed for fronto-temporal (right rostral middle frontal - left middle temporal, right caudal anterior cingulate - left middle temporal), parieto- and occipito-frontal (right cuneus - left left pars triangularis, right lateral occipital - left pars opercularis), parieto-temporal (right paracentral - left superior temporal) connections, for the left precentral - right middle temporal connection, as well as within the left temporal region (left transverse temporal - left middle temporal). Although the absolute values of these correlation values exceeded 0.4, none of them reached statistical significance after correcting for multiple comparisons; only the correlation of the post-target left transverse temporal - left middle temporal connection with reaction time was found to be marginally significant (*p* = 0.090 after correction, marked by an asterisk in Figure 5).

### 3.5 Cue-related modulation of neural entrainment

The effect of cue-related modulation on neural entrainment was investigated based on the average bandpower, ITPC, and PLV connectivity of delta-band (0.85–4 Hz) and broad-band (0.85–80 Hz) EEG signals in the post-cue period. The 80% and 50% predictable conditions were compared. In the post-cue period (excluding the time period of cue presentation) significantly larger whole-head average PLV connectivity was found in the 80% predictable condition than in the 50% predictable condition (*p* = 0.034), for the delta band. For broad-band EEG, no statistically significant difference was found between conditions in PLV connectivity. For average bandpower, no statistically significant difference was found between conditions in any of the two frequency bands, either for individual channels or for the average of all channels (*p* > 0.05, all). For ITCP, no significant clusters were identified in any of the two frequency bands.

## 4 Discussion

The present study investigated how large-scale brain networks support temporal prediction and target evaluation during auditory perception by examining EEG-based functional connectivity patterns that capture the directed temporal structure of predictability differences. By manipulating the predictability of target timing, we identified distinct cortical hubs engaged during prediction versus evaluation, shedding light on the dynamics of predictive processing. Consistent with models of neural entrainment and predictive coding (Schroeder and Lakatos, 2009; Arnal and Giraud, 2012; Benedetto et al., 2021), our results show that temporal prediction engaged a distributed fronto–temporo–parietal network, with prominent hubs in the left inferior and middle frontal gyri, superior temporal cortex, supramarginal gyrus, and insula during the post-cue prediction interval. These hubs, particularly within the frontoparietal control network, showed connectivity patterns that correlated with behavioral facilitation (i.e., faster reaction times), suggesting that temporal expectation enhances sensory readiness via top-down modulation. In contrast, post-target evaluation processing involved greater left-lateralized fronto-temporal connectivity, including the transverse temporal and middle temporal gyri, and was associated with faster responses. This dynamic shift from distributed prediction to targeted evaluation suggests that temporal prediction is not merely a function of local oscillatory entrainment. Instead, it emerges from coordinated activity across multiple large-scale networks that adapt to task demands. Predictive processing may rely on dynamic reorganization driven by prediction signals from frontal anticipation areas rather than solely on distributed sensory updating and association areas.

### 4.1 Predictive timing accelerates behavior

The present findings demonstrate that temporal prediction significantly improves response speed, aligning with theories of perceptual prediction that suggest the brain optimizes processing efficiency when event timing is predictable. Replicating the results of Stefanics et al. (2010), reaction times (RTs) were inversely related to target-onset uncertainty. Early targets preceded by an 80% predictive cue, and late targets (100% predictable once the early window elapsed), elicited markedly faster RTs compared to early targets in the 50% predictable condition. These findings align with behavioral work showing that temporal certainty sharpens perceptual readiness (Bar, 2007; Jones, 2018) and indicate that participants continuously update temporal priors when early events fail to occur (Bubic et al., 2010).

Additionally, the results suggest that participants dynamically updated their temporal expectations. In trials where an early target did not appear, participants shifted their expectation toward the late target, yielding similar RTs for late targets and early targets predicted with high confidence. This finding highlights the brain’s ability to adapt to environmental probabilities and optimize sensory processing based on updated temporal information. Overall, these findings provide strong evidence for the impact of temporal predictive processing on target detection, showing that the brain’s ability to form temporal predictions enhances sensory efficiency and responsiveness. The significant differences in RTs between conditions of varying predictability emphasize the importance of temporal cues in guiding attention and preparing the sensory system for imminent events (Jones, 2018; Large and Jones, 1999).

### 4.2 Event-related dynamics during target detection

The ERP results show that implicit temporal expectancy mainly modulates post-target processing in a distributed fronto-parietal network. A robust cluster from ∼100–500 ms after target onset exhibited more positive parietal activity and more negative frontal activity for 50% relative to 80% predictable targets, whereas no reliable cue-locked differences were detected within the cue–target interval. This pattern suggests that when temporal predictions are weak, later evaluative and decision-related processes require a greater computational load than early sensory gain mechanisms.

Early sensory components (N1, ∼90–130 ms; P2, ∼150–230 ms) typically reflect sensory gain and perceptual consolidation and can be enhanced under explicit temporal preparation (Schroeder and Lakatos, 2009; Polich, 2003, 2007). In our implicit design, however, these deflections showed no reliable modulation, consistent with reports of mixed or absent N1 effects under probabilistic timing in both audition and vision (Rimmele et al., 2011). This supports the view that implicit temporal prediction operates mainly via oscillatory phase alignment rather than large early ERP amplitude shifts. The less predictable intervals between the cue and the target likely reduced phase consistency, yielding diffuse preparatory states. Prior work suggests that low-frequency entrainment may still be functionally relevant even when subthreshold in scalp averages (Lakatos et al., 2008; Stefanics et al., 2010). Our *post hoc* analysis revealed significantly larger delta-band PLV connectivity in the 80% predictable condition than in the 50% predictable condition, supporting the importance of low-frequency neural entrainment in temporal prediction.

By contrast, later evaluative processes were more susceptible to predictability. Parietal electrodes exhibited a larger P3b for less predictable targets, consistent with context-updating accounts in which reduced temporal certainty increases model revision (Donchin and Coles, 1988; Polich, 2007). This aligns with auditory and cross-modal findings that low temporal confidence or invalid cues amplify the P3 (Rimmele et al., 2011; Jones et al., 2022). Since target acoustics were identical across conditions, these effects reflect temporal expectancy rather than sensory novelty. Frontal negativity accompanied the parietal P3b, indicating increased executive engagement when predictions were weak. Such fronto-parietal dissociations–greater parietal positivity with frontal control engagement–are typical under temporal uncertainty (Polich, 2007; Rimmele et al., 2011). Importantly, our design manipulated only temporal predictability, extending prior findings beyond joint temporal-spatial manipulations (Rimmele et al., 2011). In sum, implicit temporal expectancy reshaped target processing chiefly at later, decision-related stages. Anticipatory effects were diffuse at the scalp level, whereas parietal updating and frontal control increased under uncertainty. These findings justify moving beyond component-level ERPs to connectivity analyses that can capture distributed anticipatory information flow.

### 4.3 Functional brain networks reflecting the anticipation and detection of auditory events

This study aimed to characterize large-scale functional connectivity dynamics that support temporal prediction and subsequent target evaluation using EEG-based FRIC analysis. Our FC analysis focused on the difference waveforms between the 50% predictable and the 80% predictable conditions, but we also compared these findings with results obtained for each condition separately. This confirmed that the identified condition effects are consistent with condition-specific baseline architectures and do not reflect spurious connectivity patterns induced by waveform subtraction. The results reveal distinct but overlapping sets of cortical hubs engaged during the prediction and evaluative phases. In the post-cue prediction phase, we observed prominent connectivity hubs in either in the fontal, temporal and parietal cortices. In the post-target evaluative phase, the network of hubs became more left-lateralized, centered on the left frontal, temporal, parietal cortices. Each identified key cortical hub region during the predictive and evaluation windows is categorized according to its canonical large-scale brain network affiliation (see Table 1). Notably, we found that the strength of one post-target functional connection – between the left transverse temporal gyrus (primary auditory cortex) and the left MTG – was positively correlated with faster behavioral reaction times, albeit at a marginal level of significance. This suggests that individuals who achieved more effective coupling between early auditory regions and higher-order temporal areas were able to evaluate the target and respond more rapidly, linking stronger post-target connectivity to more efficient performance (facilitative effects of temporal orienting on behavior, Widmann and Schröger, 2022). Taken together, these findings indicate that temporal prediction engages a broad fronto-temporal-parietal network which then evolves into a more focal auditory–frontal circuit for evaluating whether the prediction was fulfilled, a pattern consistent with prior evidence of fronto-temporal interplay in auditory timing and attention tasks (Arnott et al., 2004; Rauschecker and Tian, 2000).

**TABLE 1:** Network affiliation of FRIC hub regions identified during the temporal prediction task.

Observed region (Hub)	Hemisphere	Network affiliation	Time window
Rostral middle frontal gyrus (rMFG)	Left	Frontoparietal network	Post-cue, post-target
Caudal middle frontal gyrus (cMFG)	Left	Frontoparietal network	Post-cue
Pars triangularis (IFG, BA45)	Left	Frontoparietal network	Post-cue, post-target
Pars opercularis (IFG, BA44)	Left	Frontoparietal network	Post-cue
Pars orbitalis (IFG, BA47)	Left	Frontoparietal/salience network	Post-cue
Superior temporal cortex (STG)	Bilateral	Salience network	Post-cue
Middle temporal cortex (MTG)	Bilateral	Default mode network	Post-cue, post-target
Inferior temporal cortex (ITG)	Bilateral, left	Default mode network	Post-cue, post-target
Transverse temporal cortex (Heschl’s gyrus)	Right, left	Salience network	Post-cue, post-target
Insula	Left	Salience network	Post-cue, post-target
Supramarginal gyrus (SMG)	Right	Salience/frontoparietal network	Post-cue
Precuneus	Left	Default mode network	Post-cue, post-target

Network affiliation is based on canonical large-scale functional networks described by Seeley et al. (2007). MFG, middle frontal gyrus; FPN, frontoparietal network; SN, salience network; DMN, default mode network; IFG, inferior frontal gyrus; STG, superior temporal gyrus; MTG, middle temporal gyrus; ITG, inferior temporal gyrus; SMG, supramarginal gyrus.

Importantly, the set of regions identified here maps onto known large-scale brain networks, providing clues to their functional roles in our task. The left IFG and MFG hubs are core components of the frontoparietal control network, which is recruited by cognitively demanding tasks across domains (Duncan, 2010). Consistent with their roles in executive control and working memory (Seeley et al., 2007; Uddin, 2015), these frontal regions likely served as “flexible hubs” coordinating top-down preparatory processes for temporal prediction (Cole et al., 2013; Marek and Dosenbach, 2018). Such an interpretation aligns with the Flexible Hub theory, which posits that the lateral prefrontal cortex dynamically updates its global connectivity to meet task demands (Seeley et al., 2007; Uddin, 2015). Indeed, recent evidence shows that frontoparietal regions can rapidly adjust their connectivity patterns across task states to guide behavior (Cole et al., 2013; Waskom et al., 2014). In our data, the left lateral prefrontal cortex (IFG/MFG) may serve exactly this function – interfacing with sensory regions to maintain cue-based temporal expectancy and then integrating incoming sensory evidence at target onset. In parallel, the engagement of the left insula (during both anticipatory and evaluation periods), together with the transient involvement of right SMG post-cue, points to recruitment of the salience/ventral attention network (Seeley et al., 2007; Uddin, 2015). The anterior insula is a key node of the salience network that detects behaviorally relevant events and triggers additional resource allocation (Seeley et al., 2007; Uddin, 2015). We speculate that in the predictive interval, the insula (and possibly the right temporoparietal junction region including SMG) monitored for any salient temporal cues or context changes, and at target delivery it helped mediate the switch from an internal predictive state to an external evaluation state (Corbetta and Shulman, 2002; Japee et al., 2015; Vossel et al., 2014). This is consistent with the known role of the ventral frontoparietal network in reorienting attention to unexpected or significant stimuli (Corbetta and Shulman, 2002) – here, the moment of target onset constitutes a critical event that needs to be evaluated, even if it was temporally anticipated. Finally, the consistent presence of the left precuneus (in both phases) and the involvement of lateral temporal cortices (MTG/ITG, especially post-target) may imply the default mode network (DMN) in our task. The precuneus and angular/middle temporal gyrus are established DMN nodes linked to internally generated mentation, autobiographical memory, and contextual associations (Raichle, 2015; Buckner and DiNicola, 2019). Their recruitment suggests that participants engaged brain regions responsible for maintaining an internal model of temporal context and for comparing incoming stimuli against it. In other words, DMN-mediated processes might provide a predictive “context template” against which the awaited target is evaluated. This interpretation dovetails with recent views that DMN activity can support anticipation and scene-modeling even during active tasks (Buckner and DiNicola, 2019; cf. Blank and Davis, 2016). Indeed, the involvement of MTG/ITG could reflect access to learned temporal associations or semantic expectations about the target stimulus, while the precuneus may contribute to mentally projecting the timing interval (a form of “mental time travel”; Barne et al., 2022). When the target occurred, these regions – in concert with the salience and frontoparietal regions – would participate in integrating the sensory evidence with prior expectations, leading to a “knowledge update” once the prediction outcome was clear. Such a mechanism closely parallels the concept of context updating associated with the P300 family of responses (Polich, 2007). In our data, the distributed network connectivity observed post-target (especially the link between auditory cortex and MTG that correlated with faster responses) could be viewed as the neural substrate of updating the mental model: the brain detects that the expected event has occurred (or not) and then rapidly reallocates resources to respond, consistent with P3b-like processes of evaluation and memory encoding (Polich, 2007).

Overall, our findings highlight that temporal prediction and evaluation are supported by the dynamic interactions among multiple large-scale networks rather than by any single region or pathway. This insight can be framed in the context of theoretical models of predictive brain function. On one hand, the frontal-dominant models of predictive coding propose that higher-order frontal regions generate top-down predictions that constrain sensory processing of expected inputs (Friston, 2005; Alamia and VanRullen, 2019). Our observation of prominent left prefrontal hubs during the post-cue interval accords with this view: it suggests that the brain’s predictive state was maintained by the frontal cortex, which likely sent anticipatory biasing signals to posterior regions (e.g., to the auditory cortex). Indeed, evidence from attention research shows that top-down oscillatory activity can be initiated in the frontal cortex and propagate posteriorly to modulate sensory areas (Alamia et al., 2023), providing a mechanistic basis for frontal-driven predictions. On the other hand, our data equally support distributed and hybrid integration models of predictive processing (Kok et al., 2012; Hohwy, 2013; Clark, 2013). These models argue that prediction is an emergent property of interactions across hierarchical levels of the brain rather than a one-way imposition from the top. Figure 4 reinforces this interpretation by showing that the directionality of the strongest connections of the directed temporal structure of the condition effect differs between phases: during prediction, most connections converge toward inferior frontal and temporal hubs, whereas during evaluation, the dominant flow shifts outward from frontal regions to temporal cortices. This dissociation in directional flow supports the view that frontal hubs actively generate predictions, while evaluation relies more on downstream updating mechanisms. The involvement of auditory cortices, inferior parietal regions, and DMN hubs alongside the frontal cortex in our task suggests that predictive coding is implemented via a dialogue between multiple regions: frontal areas may initiate and orchestrate predictions, but sensory and associative regions concurrently generate predictions at their own level and send feedback (or prediction errors) to update the higher areas (Clark, 2013; cf. Hohwy, 2013). This cooperative view resonates with frameworks such as “hierarchical predictive coding” and the “Bayesian brain,” in which each level of the cortical hierarchy refines predictions through the reciprocal exchange of signals (Friston, 2005; Kok et al., 2012). In our results, the co-activation of the FPN, SN, and DMN during the task can be seen as evidence for such an integrative approach: the frontoparietal network provides cognitive set-maintenance and error monitoring (Duncan, 2010; Cole et al., 2013), the salience/ventral network injects flexibility by detecting deviations and redirecting attention (Seeley et al., 2007; Vossel et al., 2014), and the default mode/temporal network contributes stored knowledge and contextual guidance (Buckner and DiNicola, 2019). Notably, the coordination among these networks is likely dynamic and time sensitive. Recent studies have demonstrated that attention and prediction involve rhythmic fluctuations in neural coupling, for example, intrinsic alpha/theta-band cycles that periodically gate information flow between frontal and sensory regions (Alamia et al., 2023; Rimmele et al., 2011; Fiebelkorn and Kastner, 2019). It is tempting to speculate that the FRIC-derived connectivity changes we observed might reflect such oscillatory coordination mechanisms, in which the brain periodically synchronizes key regions at opportune moments to optimize processing of the expected target. In line with this idea, the temporal structure of our task (with fixed cue–target intervals) could engage entrainment of neural oscillations (e.g., delta/theta) that involve both higher-order networks and auditory cortex, as shown in prior work on temporal expectations (Rimmele et al., 2011; Jones, 2018). Although our analysis was not frequency-specific, the broad agreement of our findings with these diverse theoretical accounts – from frontal executive prediction to distributed network integration – underscores a hybrid perspective: the brain’s predictive machinery appears to rely on frontal “hub” regions and distributed cortical circuits working in concert to anticipate and evaluate forthcoming events (Nobre and van Ede, 2018; Deco et al., 2021).

From an auditory neuroscience standpoint, our results also enrich the discussion through the lens of the Auditory Event Representation System (AERS) framework. Winkler and Schröger (2015) proposed the extended AERS as an integrative model in which the auditory system builds hierarchical representations of sound sequences and actively predicts upcoming sounds based on both learned regularities and top-down intentions. The present findings support this framework by demonstrating that intentional temporal prediction (driven by an instructive cue) can reconfigure connections of the directed temporal structure of the condition effect within the auditory-processing network. In particular, the AERS model emphasizes that action intentions can generate expectations for specific sensory outcomes, effectively embedding predictions into the auditory stream. Recent studies have affirmed this notion: for example, Korka et al. (2022) showed that when listeners intentionally produce sounds, the brain treats the intended sound outcome as a prediction that can modify standard deviance-detection responses. In their work, making a sound “predictable” via one’s own action led to an attenuation of the mismatch negativity (MMN) and changes in the P3a, indicating that the violation of an intention-based prediction elicits a different neural response than an externally generated surprise (Widmann and Schröger, 2022). Such findings align with the idea that the auditory system incorporates the “perceptual idea” of a forthcoming sound into its processing (Winkler and Schröger, 2015). Our connectivity results mirror this principle: during the prediction phase, we observed strengthened fronto-temporal links (e.g., between the left IFG and the auditory cortex), which can be interpreted as the neural implementation of an “intended perception” – the brain setting up a template for the expected tone at a specific time. When the target tone actually arrived, connectivity between the primary auditory cortex (left primary auditory cortex) and higher auditory regions (left MTG) was not only prominent but also predictive of faster reaction times across participants. This suggests that those individuals who most effectively aligned early auditory processing with top-down predictions (via strengthened coupling to MTG, a region implicated in contextual and semantic processing of sounds) were better at evaluating the target and initiating a response. In AERS terms, we could say that a well-coupled hierarchy (from Heschl’s gyrus up to lateral temporal cortex and down again) allowed the sensory evidence to be compared to the intention-based prediction more rapidly, resulting in quicker decision and action. This finding, although modest in effect, provides an interesting link between functional connectivity and behavior, echoing the idea that successful prediction can facilitate perception (Kok et al., 2012). That top-down auditory predictions (e.g., generated by one’s own intentions or by explicit cues) can optimize the processing of expected sounds (Jones, 2018; Schroeder and Lakatos, 2009; Arnal and Giraud, 2012; Benedetto et al., 2021). In summary, interpreting our results through the AERS and related predictive coding frameworks suggests that the brain’s response to an anticipated event is not a simple reflex to a cue, but rather a complex preparatory state where frontal, temporal, and insular regions jointly embody an “if–then” model: if the target occurs at the expected time, then here is how to process it. Our empirical evidence of distinct hub configurations for prediction vs. evaluation, and the integration of an intention-based prediction into sensory processing, provides new support for these auditory prediction models (Widmann and Schröger, 2022).

While our connectivity findings align with the idea that efficient information flow supports temporal prediction, the current data offer only preliminary evidence linking these network dynamics to behavioral performance. Although exploratory correlations between connectivity strength and behavioral facilitation revealed trends in expected directions, no effects survived correction for multiple comparisons. As such, the relationship between directed connectivity and behavioral outcomes remains tentative and should be interpreted as hypothesis-generating. Future studies with larger samples and targeted behavioral measures are needed to directly test the functional relevance of specific hubs and pathways in supporting temporal expectations.

It is important to note that while our use of high-density EEG, realistic head models, and group-level FRIC analysis improves localization reliability (Michel and Brunet, 2019), fine-grained anatomical distinctions–such as between adjacent gyri–should be interpreted cautiously. Additionally, our fixed temporal structure may have induced rhythmic entrainment effects not explicitly modeled here. To refine spatial precision and validate the identified hubs and interactions, multimodal approaches (e.g., EEG–fMRI or MEG) are essential. Simultaneous EEG–fMRI has shown promising correspondence, with studies reporting ∼40% overlap between EEG- and fMRI-derived networks (Abreu et al., 2020). Future research combining these modalities could help clarify whether the observed FRIC hubs–such as in the left IFG or precuneus–align with canonical control and DMN nodes across varying predictive contexts and sensory modalities.

For the analysis of FC, selection of the post-target window was partially informed by the ERP results, whereas the post-cue window was chosen a-priori, theoretically motivated by models of temporal anticipation while matching the duration of the post-target window to ensure comparability. Although the analysis windows were defined asymmetrically, the convergence of ERP and connectivity findings argues against a trivial data-driven explanation of the observed connectivity differences across phases and supports a genuine functional distinction between prediction and evaluation phases.

While our connectivity analyses revealed patterns consistent with canonical large-scale networks such as the DMN, SN, and FPN, it is important to note that our EEG source modeling was restricted to cortical generators and did not include subcortical structures. As such, all references to these networks should be interpreted as referring only to their cortical components. Subcortical regions–including the thalamus, basal ganglia, and hippocampus–are known to play key roles in predictive coding, salience detection, and temporal control, and are likely to interact with the cortical hubs identified here. However, given the limited spatial resolution of EEG for deep sources and the challenges of modeling subcortical activity with high reliability, we focused our analysis on cortical dynamics. Future work combining EEG with imaging modalities more sensitive to subcortical activity (e.g., MEG, fMRI, or invasive recordings) may help elucidate the full cortico-subcortical architecture of temporal prediction.

In conclusion, this study provides a comprehensive characterization of the brain’s large-scale network dynamics during the prediction and evaluation of a timed auditory event. We identified how multiple neural networks – including the frontoparietal control network, salience network, and default mode network – jointly orchestrate temporal predictions and interpret incoming sensory information. The left frontal cortex emerged as a significant hub for setting up predictions, in line with frontal-centric theories of predictive coding. Nevertheless, the concurrent engagement of temporal and parietal regions underscores that predictive processing is distributed across the brain’s hierarchy. This integrated perspective bridges theoretical frameworks from the predictive coding literature (Friston, 2005; Clark, 2013) with empirically observed network patterns, suggesting that cognitive brain networks operate in concert to minimize uncertainty about the future. Moreover, our findings extend auditory prediction models (Winkler and Schröger, 2015) by showing that intention-based expectations can modulate inter-regional connectivity and potentially facilitate behavior. While limitations of EEG source localization warrant cautious interpretation of the anatomical specifics, the overall convergence of our results with known functional networks and predictive processing models attests to the value of EEG connectivity mapping in cognitive neuroscience. The present findings advance our understanding of how the human brain proactively prepares for and evaluates events in time, revealing a tightly integrated network architecture that enables us to generate predictions, detect their outcomes, and update our internal models accordingly.

## Data Availability

The datasets presented in this study can be found in online repositories. The names of the repository/repositories and accession number(s) can be found below: Open Science Framework: https://osf.io/sa7zf/.

## Footnotes

1
https://www.nitrc.org/projects/bnv/

## Appendix

Mathematical formulations of the NDTE framework and the FRIC method

The NDTE flow *F*_*XY*_ from time series *X* to *Y* (from a source brain area to another target brain area) is defined as

F=X⁢YI(Y;i+1X|iY)i/I(Y;i+1X,iY)i
(1)

where the mutual information *I(Y*_*i* + 1_ ; *X*^*i*^|*Y*^*i*^*)* corresponds to the degree of statistical dependence between the past of *X* and the future of *Y*, and *I(Y*_*i* + 1_; *X^i^*, *Y*^i^*)* corresponds to the joint mutual information that the past of both signals together, *X* and *Y*, have about the future of *Y*. Normalizing by *I(Y*_*i* + 1_; *X^i^*, *Y^i^)* enables one to compare and combine directed mutual information flows across different pairs of brain regions. With this notation, *Y*_*i* + 1_ denotes the activity level of brain area *Y* at time point *i* + 1, and *X^i^* denotes the whole activity level of the past of *X* in a time window of length *T* up to and including time point *i* (that is, *X^i^ = [*X*_*i*_ X*_*i* – 1_ … *X*_*i* – (*T* – 1)_*])*. Following Deco et al. (2021), we estimated the value of *T* (order, or maximum lag) based on the decay to the first minimum of the autocorrelation function across conditions and participants.

*I(Y*_*i* + 1_; *X^i^*|*Y^i^)* can be calculated based on conditional entropies:

I(Y;i+1X|iY)i=H(Y|i+1Y)i-H(Y|i+1X,iY)i
(2)

where the conditional entropies are defined as follows:

H(Y|i+1Y)i=H(Y,i+1Y)i-H(Y)i
(3)

H(Y|i+1X,iY)i=H(Y,i+1X,iY)i-H(X,iY)i
(4)

and the conditional entropies can be estimated based on covariance matrices of *X* and *Y* (Brovelli et al., 2015). *I(Y*_*i* + 1_; *X^i^*, *Y^i^)* is the sum of the predictability of *Y*_*i* + 1_ by the past of *X^i^*|*Y^i^* and the internal predictability of *Y*_*i* + 1_, that is *I(Y*_*i* + 1_;*Y^i^)*:

I(Y;i+1X,iY)i=I(Y;i+1Y)i+I(Y;i+1X|iY)i
(5)

As the first step of the FRIC analysis, standardized NDTE flow values were averaged across trials and participants, resulting in a single standardized NDTE matrix of dimensions ROI × ROI. Following the notations of Deco et al. (2021), this matrix is denoted by *C*_*All*_. For each ROI *i* of *C*_*All*_, the total inflow from all ROIs of the cortical parcellation is defined as the sum of connectivity across all columns of the matrix: *G_*in*_(i)* = Σ_*j*_C_*All*(_i,j_)_. The total outflow per ROI *i* is defined as the sum of connectivity across all rows of the matrix: *G_*out*_(i)* = Σ*_*j*_C_*All(j,i)*_*.

The major hubs were defined through an iterative process. After sorting the regions by inflow from highest to lowest, the largest subset of ROIs *k* was found that had a value *G_*FRIC*_(k)* significantly larger than any other set with the same number of regions. *G_*FRIC*_(k)* is defined as follows:

G(k)F⁢R⁢I⁢C=ΣkCA⁢l⁢l⁢(k)+ΣkGi⁢n(k)-ΣkGo⁢u⁢t(k)
(6)

where Σ*_*k*_C_*All(k)*_* is the sum of connections between all club members, Σ*_*k*_G_*in*_(k)* is the sum of all incoming connections of the club members, and Σ*_*k*_G_*out*_(k)* is the sum of all outgoing connections of the club members. The significance value of each *G_*FRIC*_(k)* was assessed via 100000 Monte Carlo simulations. In each permutation, surrogate clubs with *k* members were created that had the same *k* − 1 members with one added random new member. Starting with the ROI having the largest incoming NDTE flow, the algorithm continued to confirm FRICs as long as the *p*-value of the comparison between the considered FRIC and surrogate clubs was smaller than 0.05.
